# Effects of Probiotic and Selenium Co-supplementation on Lipid Profile and Glycemia Indices: A Systematic Review and Meta-analysis of Randomized Clinical Trials

**DOI:** 10.1007/s13668-023-00448-1

**Published:** 2023-02-13

**Authors:** Vida Mohammadparast, Tanin Mohammadi, Elham Karimi, Beth L. Mallard

**Affiliations:** 1 School of Health Sciences, Massey University, PO Box 756, Wellington, 6021 New Zealand; 2 Department of Clinical Nutrition, School of Nutrition and Food Science, Food Security Research Center, Isfahan University of Medical Sciences, Isfahan, Iran

**Keywords:** Probiotics, Selenium, Lipid profile, Glycemia indices

## Abstract

**Purpose of Review:**

The current systematic review and meta-analysis was done to evaluate the effects of selenium and probiotic co-supplementation on lipid profile and glycemia indices of the adult population using randomized controlled clinical trials (RCTs).

**Recent Findings:**

Five studies involving 282 participants with a sample size ranging from 38 to 79 were eligible to be enrolled in the current study. Co-supplementation with probiotic and selenium reduced fasting plasma glucose (WMD =  −4.02 mg/dL; 95% CI: −5.87 to −2.18; *P* < 0.001), insulin (WMD =  −2.50 mIU/mL; 95% CI: −3.11 to −1.90; *P* < 0.001), homeostatic model assessment for insulin resistance (WMD =  −0.59; 95% CI: −0.74 to −0.43; *P* < 0.001), quantitative insulin sensitivity check index (WMD = 0.01; 95% CI: 0.01 to 0.02; *P* < 0.001), total cholesterol (WMD =  −12.75 mg/dL; 95% CI: −19.44 to −6.07; *P* < 0.001), low-density lipoprotein cholesterol (WMD =  −7.09 mg/dL; 95% CI: −13.45 to −0.73; *P* = 0.029), and triglyceride (WMD =  −14.38 mg/dL; 95% CI: −23.13 to −5.62; *P* = 0.001).

**Summary:**

The findings of the current systematic review and meta-analysis suggested that co-supplementation with probiotics and selenium may benefit adults in terms of glycemia indices and lipid profile. However, due to the small number of included studies, further trials are needed to confirm our findings.

**Supplementary Information:**

The online version contains supplementary material available at 10.1007/s13668-023-00448-1.

## Introduction

With nearly one in three deaths, cardiovascular disease (CVD) is most common cause of death in the USA [1]. Most of the CVD risk factors are either modifiable or preventable including cigarette smoking, overweight/obesity, diabetes, dyslipidemia, and high blood pressure [2]. Accordingly, 14% of US adults smoke cigarettes [2], 72% are overweight/obese [3], 14% have diabetes [4], 29% have hypercholesterolemia [3], and 32% have hypertension [5]. Moreover, the American Heart Association predicted an increase in CVD prevalence accompanied by health care costs by 2030 [6]. Therefore, finding effective approaches to prevent or modify CVD risk factors should be prioritized.

It has been reported that gut microbiota, microbes living in the human intestinal tract, may affect CVD pathogenesis and related risk factors [7]. Early studies on the gut microbiome proposed that alteration of the composition of the fecal microbial community is linked with insulin resistance and obesity [8, 9]. Moreover, sequencing studies also suggested an association between gut microbiota and atherosclerosis [10]. Therefore, strategies to improve the composition of gut microbiota are suggested as a complementary approach for preventing CVD through modifying related risk factors.

Probiotics are defined as living microorganisms that exert beneficial health effects when consumed in adequate amounts. It has been suggested that joint selenium and probiotic supplementation are much more effective than single selenium or probiotic supplementation in terms of metabolic profile [11•]. Moreover, co-supplementation of selenium and probiotics in animal studies also suggested a synergistic effect on metabolic profile compared to the selenium or probiotics alone [12, 13]. To date, various clinical trials have been conducted to evaluate the effects of probiotic and selenium co-supplementation on CVD risk factors; however, their findings are contradictory [11•, 14, 15••, 16, 17]. Moreover, their sample size is small which precludes clinicians to reach a firm conclusion in this regard. Therefore, the current systematic review and meta-analysis was done to evaluate the effects of selenium and probiotic co-supplementation on lipid profile and glycemia indices of the adult population using randomized controlled clinical trials (RCTs).

## Methods

### Search Strategy and Data Source

Selected electronic databases including ISI Web of Science, PubMed, and Scopus were searched systematically from the earliest available date to February 2022 to find relevant studies. Two independent reviewers conducted a database search to identify studies that investigated the effects of selenium and probiotic co-supplementation on lipid profile and glycemia indices using the following keywords: probiotic OR probiotics OR Lactobacillus OR Bifidobacterium OR Streptococcus OR Saccharomyces OR Enterococcus AND selenium (Table 1). The reference list of eligible studies was also screened to minimize the chance of missing relevant studies. Since the studied outcomes in the present study may have been considered a secondary outcome in the primary studies and therefore not mentioned in the abstract, we conducted a systematic search without considering these outcomes (i.e., lipid profile and glycemia indices) and then examined them in the title/abstract and full-text phases. No filtering was made upon the database searching in terms of publication time, study design, and language. The present study was done on the basis of the Preferred Reporting Items for Systematic Reviews and Meta-Analysis (PRISMA) statements [18].

**Table 1:** Search strategy of selected databases

**PubMed**
Search hits: 377
("probiotic"[Title/Abstract] OR "probiotics"[Title/Abstract] OR "Lactobacillus"[Title/Abstract] OR "Bifidobacterium"[Title/Abstract] OR "Streptococcus"[Title/Abstract] OR "Saccharomyces"[Title/Abstract] OR "Enterococcus"[Title/Abstract]) AND ("selenium"[Title/Abstract]))

**Scopus**
Search hits: 1256
((TITLE-ABS-KEY(probiotic) OR TITLE-ABS-KEY(probiotics) OR TITLE-ABS-KEY(lactobacillus) OR TITLE-ABS-KEY(bifidobacterium) OR TITLE-ABS-KEY(streptococcus) OR TITLE-ABS-KEY(saccharomyces) OR TITLE-ABS-KEY(enterococcus))) AND (TITLE-ABS-KEY(selenium))

**ISI Web of Science**
Search hits: 688
(TOPIC: (probiotic) OR TOPIC: (probiotics) OR TOPIC: (lactobacillus) OR TOPIC: (bifidobacterium) OR TOPIC: (streptococcus) OR TOPIC: (saccharomyces) OR TOPIC: (enterococcus)) AND (TOPIC: (selenium))

Lowercase letters in the same column indicate significant differences among soil depths, while uppercase letters indicate significant differences between M3 and M6. All differences were considered to be significant at *p* < 0.05.

### Eligibility Criteria and Study Selection

The PICOS (Population, Intervention, Comparison, Outcome, Study design) framework was used during study selection as follows: P (> 18 years individuals), I (probiotic + selenium supplement), C (placebo), O (lipid profile [triglyceride (TG), high-density lipoprotein cholesterol (HDL-C), low-density lipoprotein cholesterol (LDL-C), total cholesterol (TC)] and glycemia indices [homeostatic model assessment for insulin resistance (HOMA-IR), quantitative insulin sensitivity check index (QUICKI), fasting insulin, and fasting plasma glucose (FPG)]), S (RCTs). All the search results were exported to the EndNote X7 software (Thomson Corporation, Stamford, USA) to be screened by two independent investigators for eligible studies. Inclusion criteria were as follow: original peer-reviewed full-text RCTs recruited > 18 years old subjects with either parallel or cross-over design that implemented co-supplementation of selenium and probiotic and assessed at least one of the outcomes of interest. Exclusion criteria were as follows: non-human studies; recruited < 18 years old individuals; or non-original full-length studies (i.e., review articles, poster abstract, commentary, editorials, and case reports).

### Data Extraction

Eligible articles were screened by two independent reviewers for extraction of the data of interest using pre-defined Excel sheets. The extracted data were as follows: first author’s name, year of publication, study location, characteristics of the study population (e.g., sample size, sex, and body mass index [BMI]), study duration, RCT design, the dose of selenium and probiotics, number of probiotic bacteria, and mean and standard deviation (SD) of change in each outcome in the intervention and the control group.

### Risk of Bias Assessment

The risk of bias of the included studies was examined using the Cochrane Collaboration’s tool [19]. It consists of seven domains including random sequence generation, allocation concealment, blinding of participants and personnel, blinding of outcome assessments, incomplete outcome data, selective reporting, and other biases. Each domain scored as low risk, high risk, or unclear risk, and the overall risk of bias for each study was stated as good, fair, and poor quality.

### Statistical Analysis

Statistical analysis was done using STATA software (version 11.0; Stata Corporation). For each outcome, data was collected as mean ± SD in a similar unit to estimate the pooled effect size. Weighted mean differences (WMDs) with corresponding 95% confidence intervals (CIs) were calculated for each studied outcome using inverse-variance fixed-effect models. Between effect sizes heterogeneity was estimated using the I-squared (*I*^*2*^) index and values equal to 25, 50, or 75% were interpreted as low, moderate, or high heterogeneity, respectively. Visual inspection of funnel plots in combination with Begg’s and Egger’s tests was implemented to assess publication bias. The influence of each study on overall meta-analysis findings was examined via sensitivity analysis. Each time, one study was removed and a meta-analysis was done with the remaining articles to evaluate the robustness of the findings. *P* values < 0.05 considered statistically significant.

## Results

### Search Findings

**Fig. 1:**
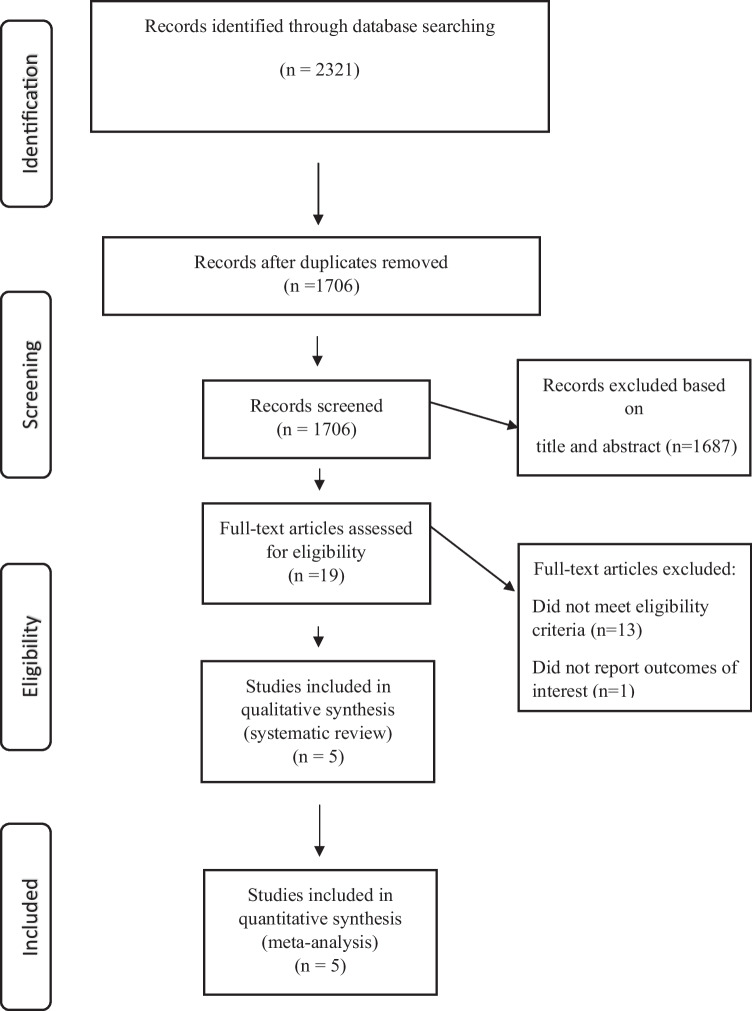
The flow diagram of study selection

A primary search of the selected electronic databases yielded a total of 2321 articles. After the omission of duplicates, 1706 studies remained to be screened on the basis of title/abstract by two independent investigators. Nineteen articles remained after this phase and subsequently were assessed on the basis of full-text and finally, five documents were selected to be eligible for the current systematic review and meta-analysis. The PRISMA flow diagram of the selection process is shown in Fig. 1.

### General Characteristics of the Included Studies

Five studies involving 282 participants with a sample size ranging from 38 to 79 were eligible to be enrolled in the current systematic review and meta-analysis regarding the beneficial role of probiotics and selenium co-supplementation on parameters of glycemia and lipid profile. Participants’ mean age and BMI at baseline ranged from 27.2 to 77.8 years and 21.13 to 30.65 kg/m^2^. The included studies were conducted between 2005 and 2021 in Iran [11•, 15••, 16, 17] and Slovakia [14]. All of the included studies were double-blind with an intervention duration ranging from 12 to 60 weeks. Enrolled studies administered selenium with a dose ranging from 50 to 200 µg/day in combination with a probiotic supplement with a number of strains ranging from 1 to 4. The general characteristics of the included studies are presented in Table 2.

**Table 2:** Characteristics of the included studies

**First author (publication year)**	**Country**	**Total sample size** **(M/F)**	**Target population**	**Mean age (year)**	**Mean BMI (kg/m**^**2**^**)**	**RCT design (blinding)**	**Duration (weeks)**	**Intervention of experimental group (Dose)**	**Number of bacteria**	**Intervention of control group**	**ROB**
Tamtaji et al. (2019)	Iran	79	Alzheimer’s disease	77.8	21.13	Parallel (yes)	12 weeks	Selenium (200 mg/day) plus probiotic containing *L. acidophilus*, *B. bifidum*, and *Bifidobacterium longum* (6 × 10^9^ CFU/day)	3	Placebo	Good
Hlivak et al. (2005)	Slovakia	7/31	Elderly nursing home population	76.7	29.24	Parallel (yes)	60 weeks	Selenium (50 µg/day) plus probiotic containing *Enterococcus faecium* (2 × 10^9^ CFU/day)	1	Placebo	Fair
Raygan et al. (2019)	Iran	21/33	Patients with T2DM and CHD	63.6	30.65	Parallel (yes)	12 weeks	Selenium (200 µg/day) plus probiotic containing *Lactobacillus acidophilus*, *Lactobacillus reuteri*, *Lactobacillus fermentum*, and *Bifidobacterium bifidum* (8 × 10^9^ CFU/day)	4	Placebo	Good
Jamilian et al. (2021)	Iran	51	Chronic Schizophrenia	45.1	24.8	Parallel (yes)	12 weeks	Selenium (200 µg/day) plus probiotic containing *Lactobacillus acidophilus*, *Lactobacillus reuteri*, *Lactobacillus fermentum*, and *Bifidobacterium bifidum* (8 × 10^9^ CFU/day)	4	Placebo	Good
Shabani et al. (2018)	Iran	60 F	Polycystic Ovary Syndrome	27.2	25.15	Parallel (yes)	12 weeks	Selenium (200 µg/day) plus probiotic containing *Lactobacillus acidophilus*, *Lactobacillus reuteri*, *Lactobacillus fermentum*, and *Bifidobacterium bifidum* (8 × 10^9^ CFU/day)	4	Placebo	Good

*M* male, *F* female, *BMI* body mass index, *RCT* randomized controlled trial, *ROB* risk of bias, *T2DM* type 2 diabetes mellitus, *CHD* coronary heart disease, *CFU* colony-forming unit

### Risk of Bias of the Included Studies

The findings of the risk of bias of the included studies are shown in Table 2. As can be seen, all of the enrolled studies were low risk in terms of blinding of participants and personnel, blinding of outcome assessment, incomplete outcome data, selective reporting, and other sources of bias. For domains of random sequence generation and allocation concealment, all of the studies were low risk except for the work of Hlivak et al. which was unclear. Overall, four studies [11•, 15••, 16, 17] ranked as high quality and one [14] as fair quality.

### Findings from Meta-analysis

#### The Effect of Probiotic and Selenium Co-supplementation on FPG

**Fig. 2:**
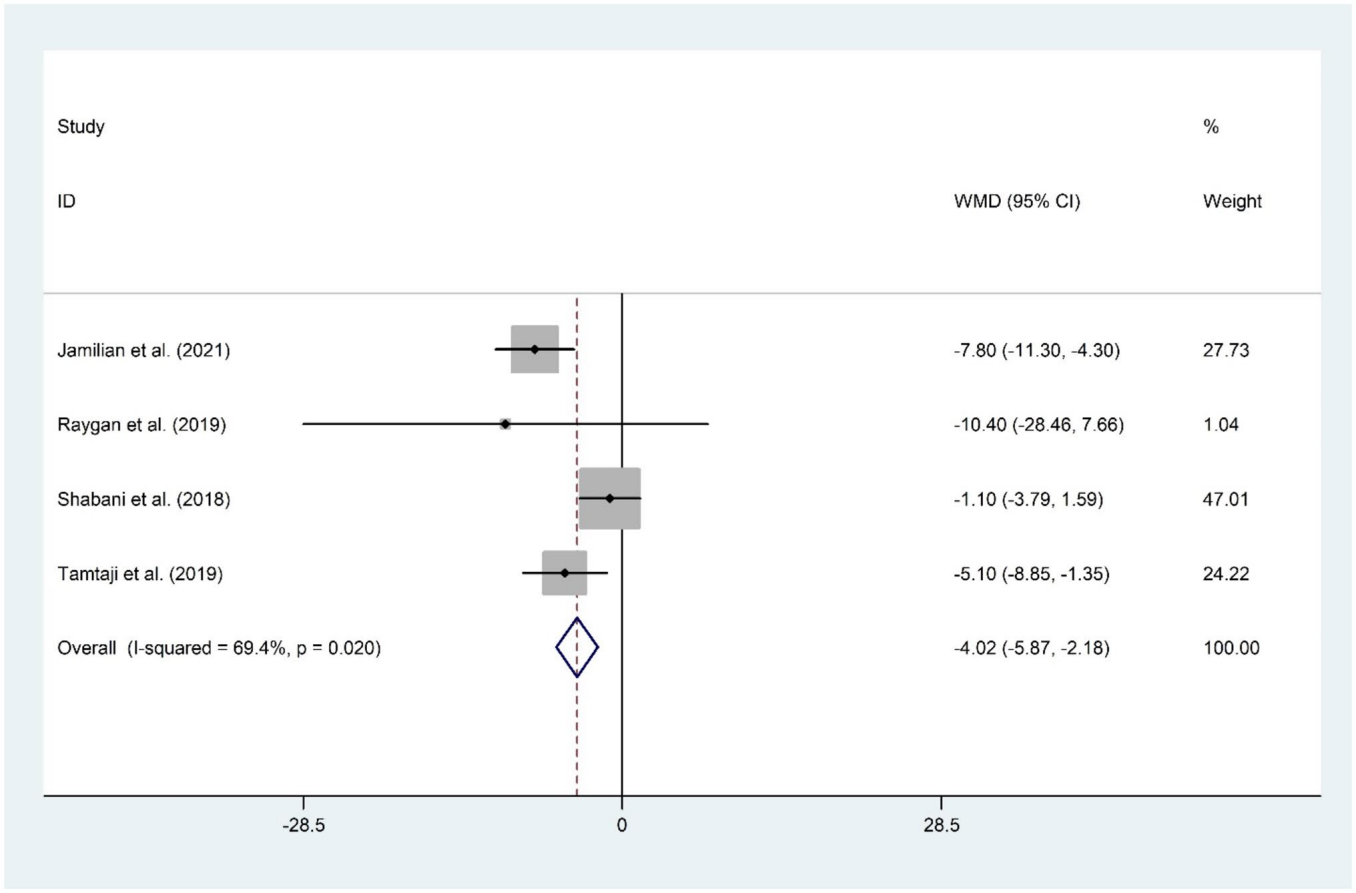
Forest plot of the effect of probiotic and selenium co-supplementation on FPG

Co-supplementation of probiotic and selenium was examined using four datasets [11•, 15••, 16, 17] including 244 subjects. Overall meta-analysis revealed a beneficial role for probiotic and selenium in reducing FPG (WMD =  −4.02 mg/dL; 95% CI: −5.87 to −2.18; *P* < 0.001) with evidence of a significant heterogeneity (*I*^2^ = 69.4%, *P* = 0.020). The overall finding was not sensitive to any individual study. No evidence of publication bias was observed (*P* = 0.497, Begg’s test, and *P* = 0.539, Egger’s test) (Fig. 2).

#### The Effect of Probiotic and Selenium Co-supplementation on Insulin

**Fig. 3:**
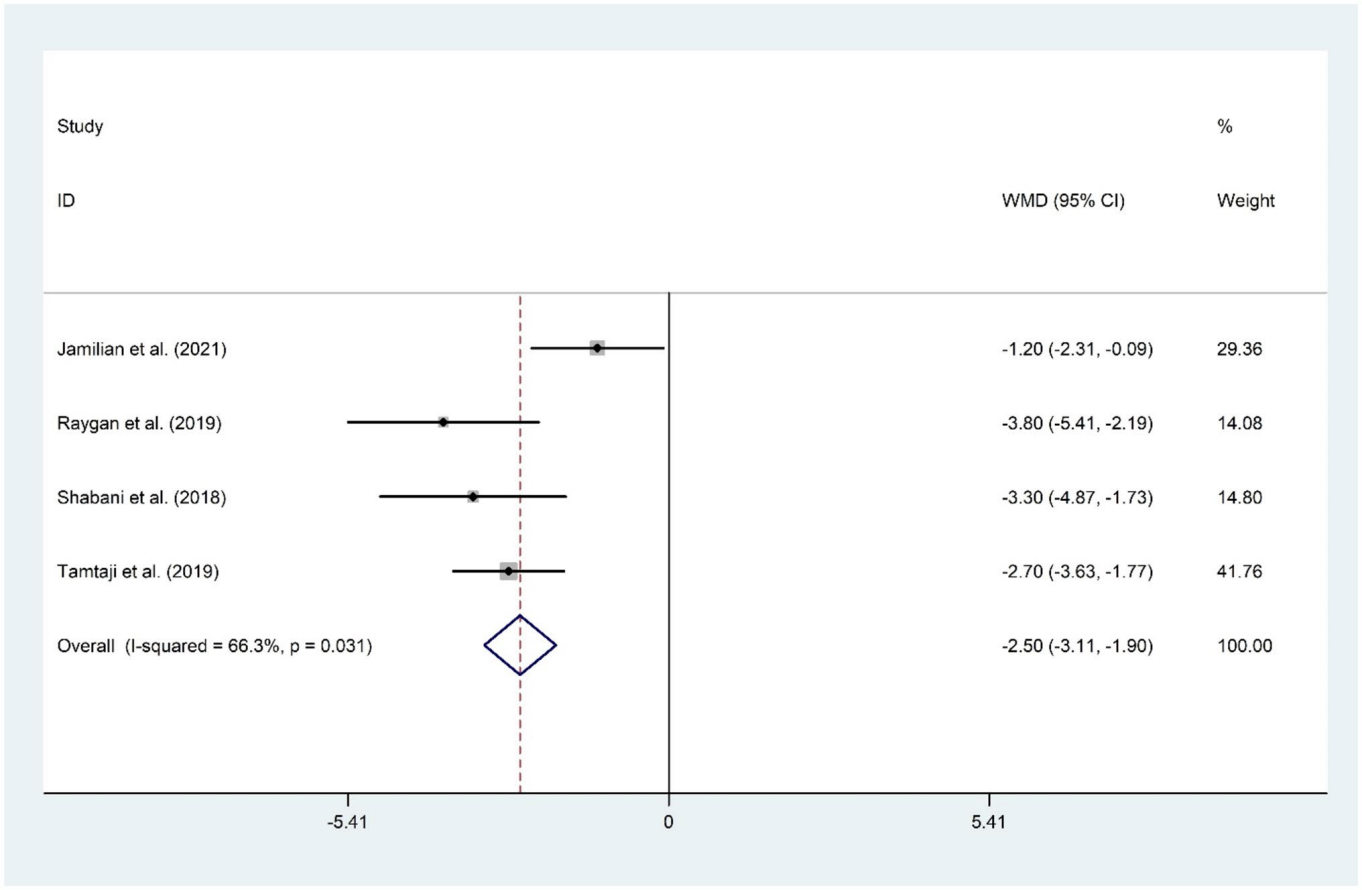
Forest plot of the effect of probiotic and selenium co-supplementation on insulin

The analysis of four studies [11•, 15••, 16, 17] regarding the effect of probiotic and selenium co-supplementation in serum levels of insulin proposed a significant reduction (WMD =  −2.50 mIU/mL; 95% CI: −3.11 to −1.90; *P* < 0.001). There was evidence of significant heterogeneity among the included studies (*I*^2^ = 66.3%, *P* = 0.031). The overall result was not changed following sensitivity analysis. No evidence of publication bias was observed (*P* = 0.174, Begg’s test, and *P* = 0.486, Egger’s test) (Fig. 3).

#### The Effect of Probiotic and Selenium Co-supplementation on HOMA-IR

**Fig. 4:**
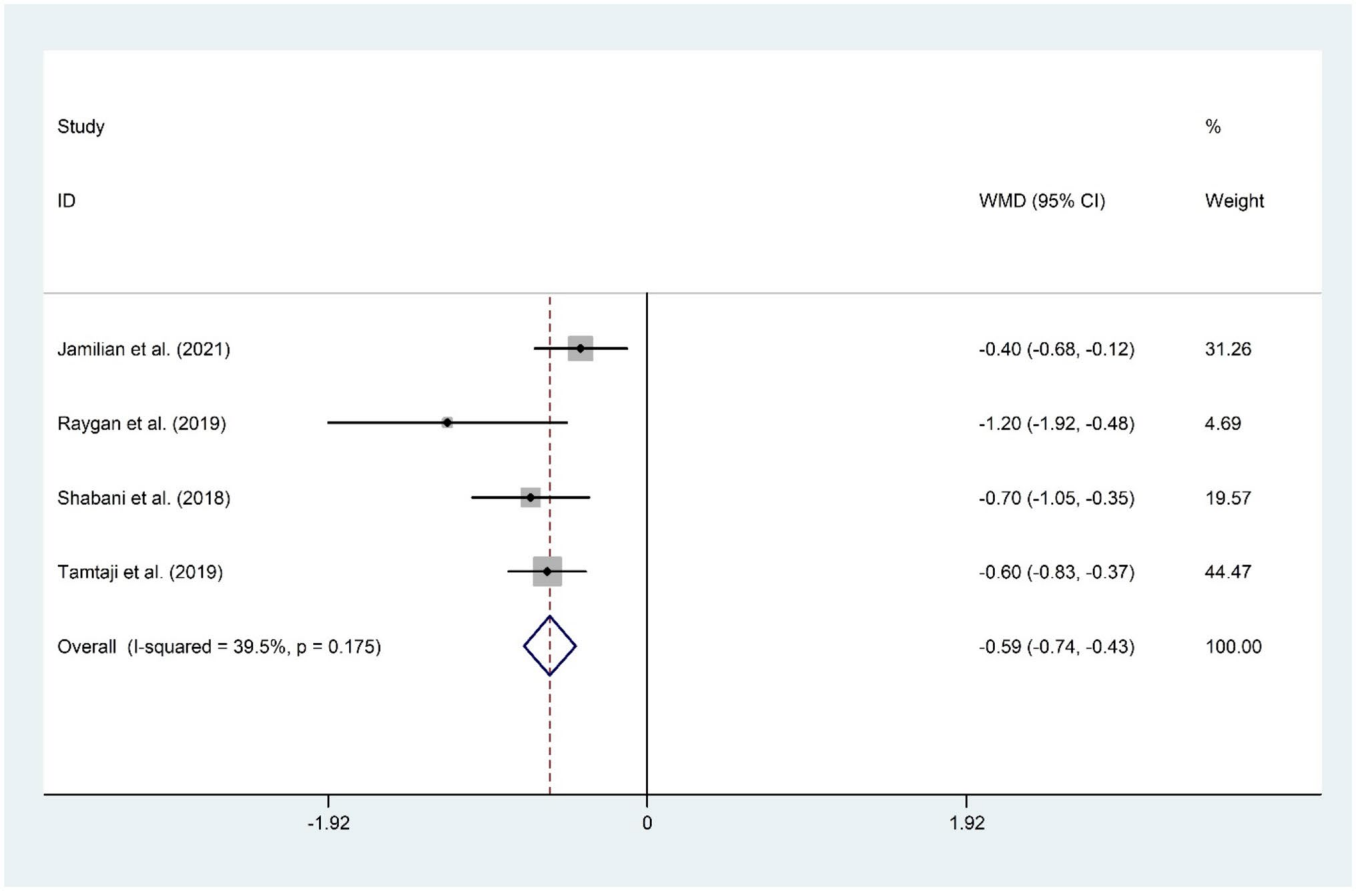
Forest plot of the effect of probiotic and selenium co-supplementation on HOMA-IR

The meta-analysis of four documents [11•, 15••, 16, 17] for the mean differences in HOMA-IR suggested a significant reduction following probiotic and selenium co-supplementation (WMD =  −0.59; 95% CI: −0.74 to −0.43; *P* < 0.001) with evidence of moderate heterogeneity (I2 = 39.5%, *P* = 0.175). The overall result was not sensitive to any individual study. No evidence of publication bias was observed (*P* = 0.174, Begg’s test, and *P* = 0.234, Egger’s test) (Fig. 4).

#### The Effect of Probiotic and Selenium Co-supplementation on QUICKI

**Fig. 5:**
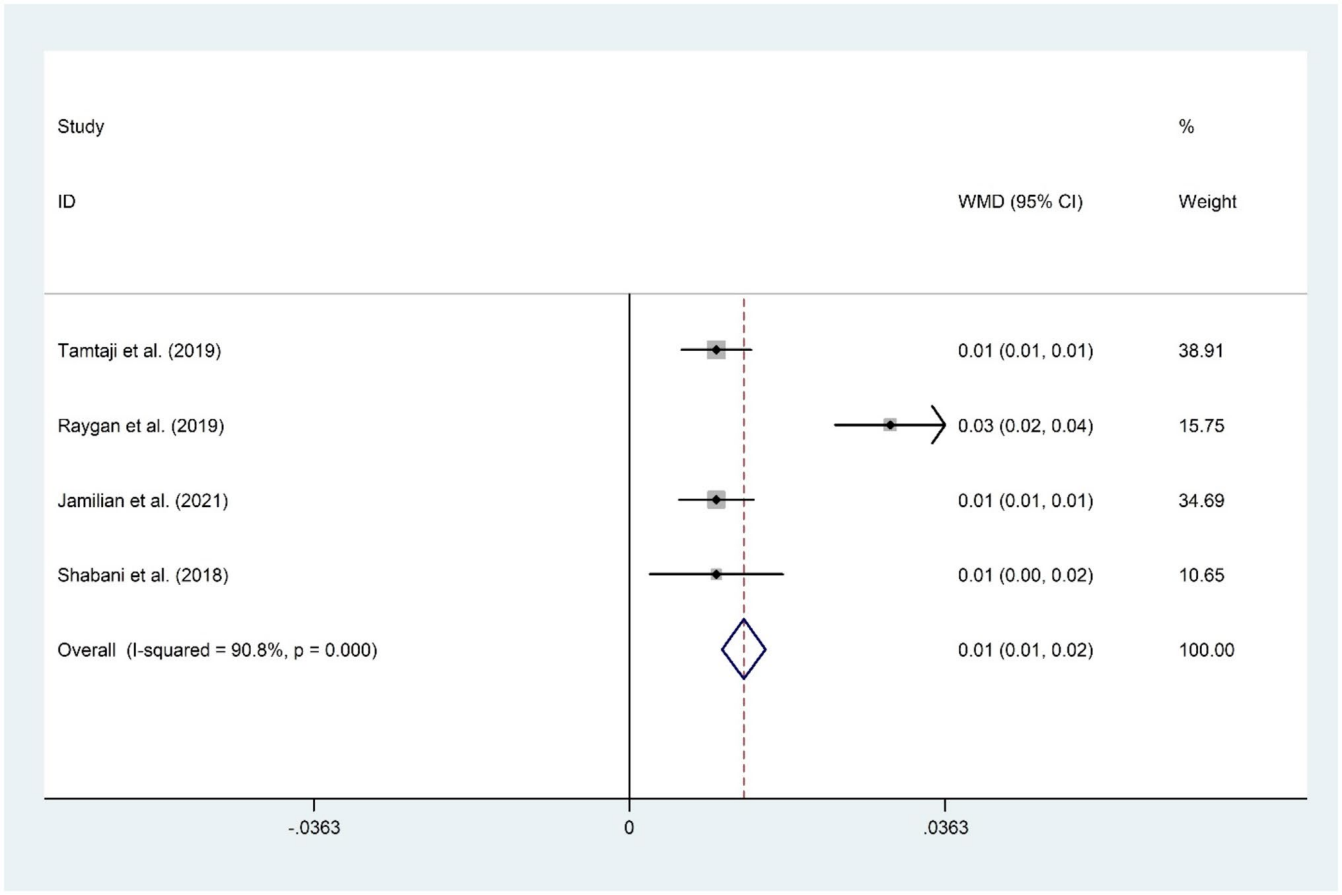
Forest plot of the effect of probiotic and selenium co-supplementation on QUICKI

The effect of probiotic and selenium co-supplementation on QUICKI was assessed by four RCTs including 244 individuals [11•, 15••, 16, 17]. Overall meta-analysis revealed that probiotic and selenium co-supplementation significantly improve QUICKI (WMD = 0.01; 95% CI: 0.01 to 0.02; *P* < 0.001) with evidence of a considerable heterogeneity (*I*^2^ = 16.8%, *P* = 0.307). The overall finding was not sensitive to any individual study. No evidence of publication bias was observed (*P* = 0.174, Begg’s test, and *P* = 0.497, Egger’s test) (Fig. 5).

#### The Effect of Probiotic and Selenium Co-supplementation on TC

**Fig. 6:**
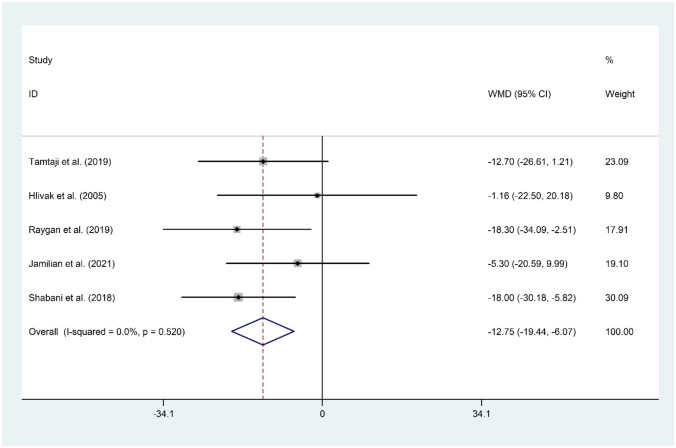
Forest plot of the effect of probiotic and selenium co-supplementation on TC

Five datasets [11•, 14, 15••, 16, 17] with a total sample size of 282 patients evaluated the effect of probiotic and selenium co-supplementation on serum levels of TC. The overall findings suggested a significant reduction in TC following administration of probiotic and selenium (WMD =  −12.75 mg/dL; 95% CI: −19.44 to −6.07; *P* < 0.001) with no evidence of heterogeneity (*I*^2^ = 0.0%, *P* = 0.520). Sensitivity analysis revealed that no study can influence overall meta-analysis findings. No evidence of publication bias was observed (*P* = 0.142, Begg’s test, and *P* = 0.177, Egger’s test) (Fig. 6).

#### The Effect of Probiotic and Selenium Co-supplementation on LDL-C

**Fig. 7:**
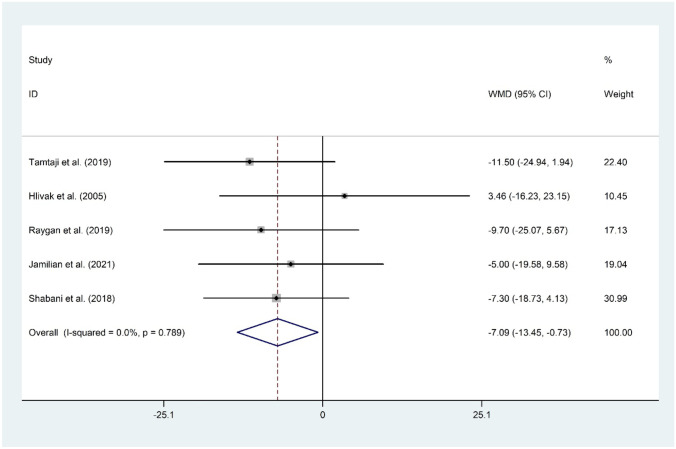
Forest plot of the effect of probiotic and selenium co-supplementation on LDL-C

The hypothesis of the beneficial role of probiotic and selenium co-supplementation on serum levels of LDL-C was evaluated in five studies [11•, 14, 15••, 16, 17] with a total sample size of 282 individuals. Overall meta-analysis revealed a significant reduction in LDL-C (WMD =  −7.09 mg/dL; 95% CI: −13.45 to −0.73; *P* = 0.029) with no evidence of heterogeneity (*I*^2^ = 0.0%, *P* = 0.789). The overall finding was sensitive to Raygan et al. (WMD =  −6.55 mg/dL; 95% CI: −13.53 to 0.43) and Shabani et al. (WMD =  −6.99 mg/dL; 95% CI: −14.65 to 0.66) studies. No evidence of publication bias was observed (*P* = 0.327, Begg’s test, and *P* = 0.243, Egger’s test) (Fig. 7).

#### The Effect of Probiotic and Selenium Co-supplementation on HDL-C

**Fig. 8:**
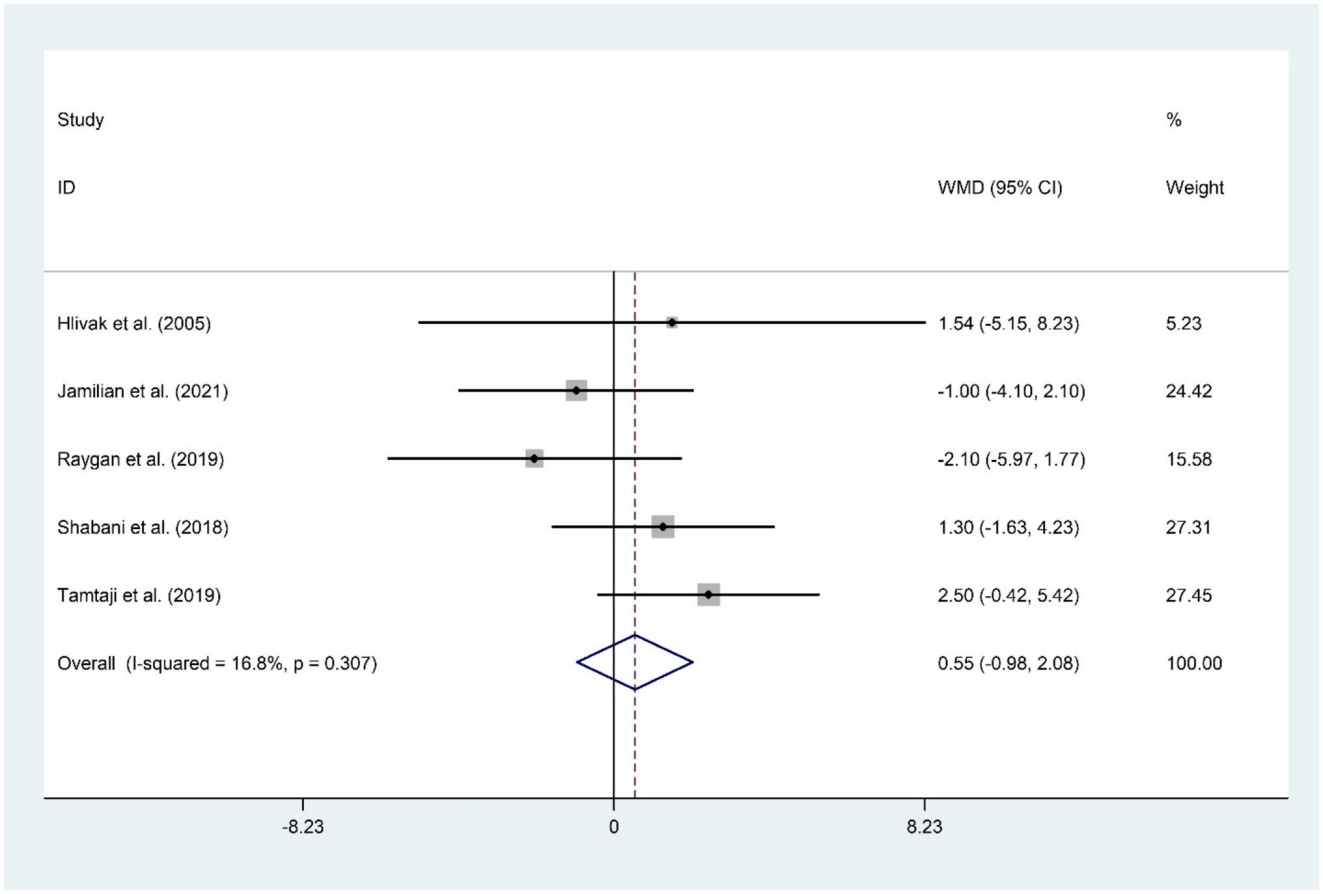
Forest plot of the effect of probiotic and selenium co-supplementation on HDL-C

Five studies [11•, 14, 15••, 16, 17] consisting of 282 participants reported on the effect of probiotic and selenium co-supplementation on HDL-C. Probiotic and selenium consumption could not improve HDL-C (WMD = 0.55 mg/dL; 95% CI: −0.98 to 2.08; *P* = 0.481) with no evidence of significant heterogeneity (*I*^2^ = 16.8%, *P* = 0.307). The overall result was not changed following sensitivity analysis. No evidence of publication bias was observed (*P* = 0.142, Begg’s test, and *P* = 0.772, Egger’s test) (Fig. 8).

#### The Effect of Probiotic and Selenium Co-supplementation on TG

**Fig. 9:**
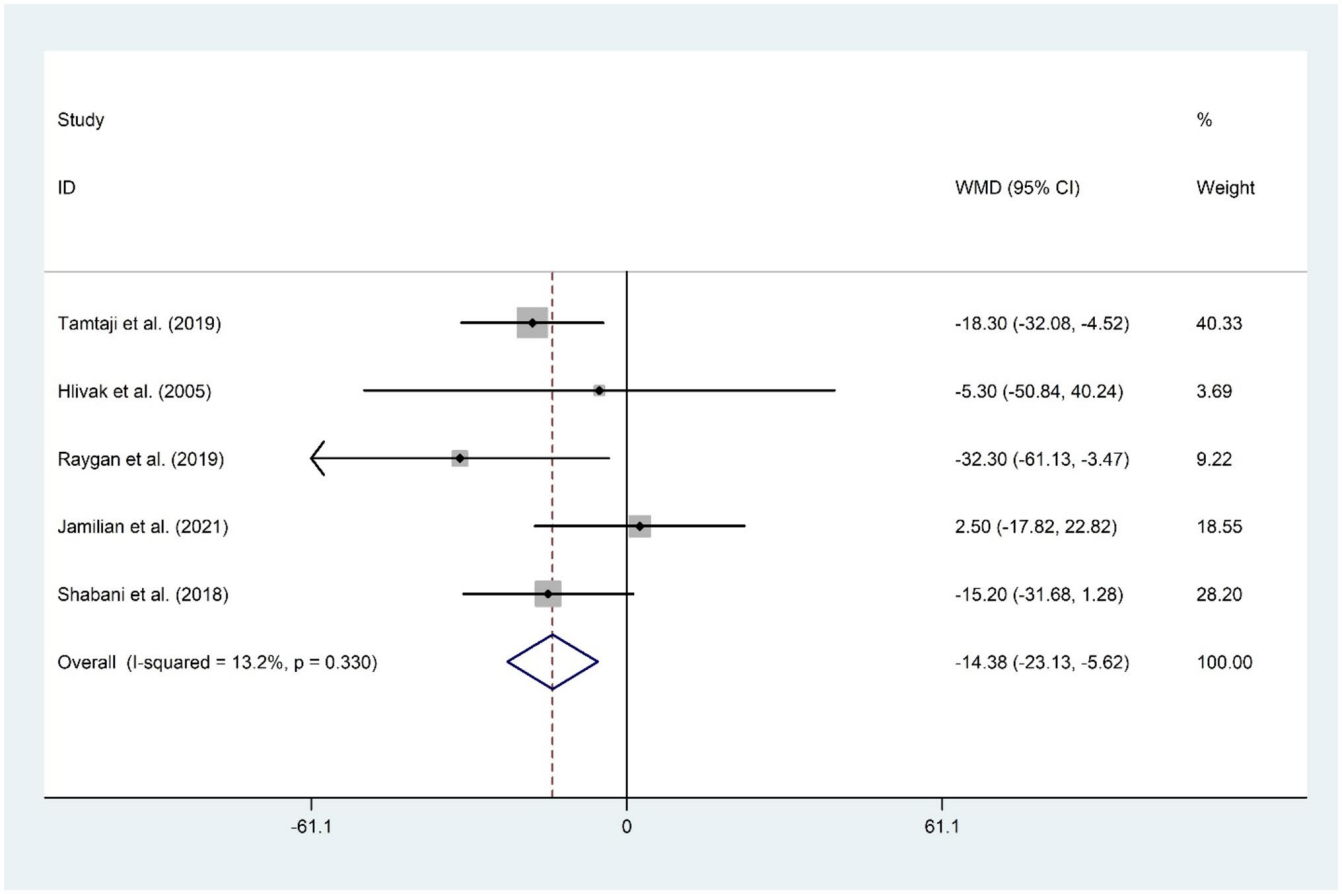
Forest plot of the effect of probiotic and selenium co-supplementation on TG

The meta-analysis of five datasets [11•, 14, 15••, 16, 17] proposed a significant decrease in serum levels of TG following probiotic and selenium co-supplementation (WMD =  −14.38 mg/dL; 95% CI: −23.13 to −5.62; *P* = 0.001). There was no evidence of significant heterogeneity among the included studies (*I*^2^ = 13.2%, *P* = 0.330). The omission of each study did not change the overall meta-analysis finding. No evidence of publication bias was observed (*P* = 0.624, Begg’s test, and *P* = 0.892, Egger’s test) (Fig. 9).

## Discussion

The current systematic review and meta-analysis was conducted to reach a firm conclusion on the role of selenium and probiotic co-supplementation on lipid profile and glycemia indices of the adult population. Our findings suggested that probiotic and selenium co-supplementation could significantly improve all the glycemia indices including FPG, insulin, HOMA-IR, QUICKI, and lipid profile parameters including TC, LDL-C, and TG. These findings may imply that co-supplementation with selenium and probiotic can be used as a complementary approach in modifying CVD risk factors; however, between-study heterogeneity should be taken into account during the interpretation of results.

The hypothesis of selenium and probiotic co-supplementation was suggested by previous animal studies that mentioned the superiority of combined probiotic and selenium compared to each of the probiotics or selenium alone [12, 13]. In agreement with these findings, Tamtaji et al. also proposed that selenium and probiotic co-supplementation show greater efficacy in terms of metabolic markers compared to probiotics alone [11•]. Synergistic effects of selenium supplementation on lipid profile and glycemia indices may be explained through its inhibitory properties on the expression of P-selectin and cyclooxygenase-2 and upregulation of some fatty acid enzymes including medium-chain Acyl-CoA dehydrogenase and very-long-chain dehydrogenase [20]. Moreover, it was observed that selenization of lactic acid bacteria strains led to binding selenium to their cells, and subsequently increase their antioxidant capacity compared to the parental strains. Integration of accumulated selenium in bacterial cells with antioxidant enzymes seemed to increase the antioxidant properties of these strains [21]. Moreover, enrichment of probiotic bacteria with selenium can affect their cell surface hydrophobicity. This factor has an important role in the adhesion of bacteria to epithelial cells, resulting in the colonization of intestinal epithelium by beneficial bacteria. The assessment of the hydrophobicity of the selenized probiotic bacteria showed higher hydrophobicity values in comparison to the parental strains [21].

In the present work, probiotic and selenium co-supplementation significantly reduced serum levels of FPG (−4.02 mg/dL), insulin (= −2.50 mIU/mL), HOMA-IR (−0.59), and increased QUICKI (0.01). It was reported among Asia Pacific region that each 18 mg/dL lower FPG was associated with a 23% lower risk of ischemic heart disease and a 21% lower risk of total stroke [22]. A meta-analysis proposed that an increase of 7.19 mIU/mL fasting insulin led to a 18% higher risk of CVD [23]. A more recent meta-analysis also revealed that each 6.26 mIU/mL, 2.23, and 18.9 mg/dL increase in insulin, HOMA-IR, and FPG correspondence to relative risk of 1.04, 1.46, and 1.21 for CVD, respectively [24]. Previous documents are in line with our findings regarding the beneficial role of probiotic supplementation on diabetes health outcomes [25, 26]. Moreover, another meta-analysis suggested that pro-/synbiotic supplementation for a duration ≥ 12 weeks can improve glycemia indices. Other meta-analyses also proposed a beneficial effect of probiotic consumption on glycemia indices [27–30]; however, uncertainties still remain regarding the best form (supplement/food) and species of probiotics [31]. Reduction in inflammatory signals and increase in T-cell receptors and hepatic natural killer receptors are among the other suggested mechanisms regarding the role of probiotics on insulin resistance [32, 33]. Also, augmented gut permeability can lead to translocation of bacterial products (inflammatory lipopolysaccharides (LPS)), leading to insulin resistance [34]. Probiotic consumption can diminish gut permeability leading to improve insulin sensitivity, as evidenced by reduced zonulin and calprotectin levels following probiotic supplementation [35, 36]. Probiotics can improve the gut microbiome that plays an important role in metabolizing bile acid subsequently leading to a reduction in insulin resistance and inflammation [37].

Results of our meta-analysis revealed a significant reduction in TC (−12.75 mg/dL), LDL-C (−7.09 mg/dL), and TG (−14.38 mg/dL) but not in HDL-C (0.55 mg/dL) following selenium and probiotic co-supplementation. A meta-analysis reported that a 1 mmol/l (38.67 mg/dL) reduction in LDL-C level linked with a 19% reduction in CVD-related mortality and a 12% reduction in all-cause mortality [38]. Our findings were in line with previous work [39–41] regarding the beneficial role of probiotics on lipid parameters’ improvement. Probiotics can reduce the reabsorption of bile cholesterol and diminish dietary cholesterol absorption by incorporating cholesterol in their cellular membrane [42]. Also, some species of probiotics can produce hydrolases leading to lower cholesterol absorption via higher bile salt excretion [43, 44]. Production of short-chain fatty acids (SCFA) (i.e., butyrate and propionate) increased following probiotic consumption which led to the inhibition of hydroxymethylglutaryl CoA reductase (HMG-CoA reductase), reducing cholesterol synthesis [45]. Moreover, it was suggested that butyrate can improve insulin sensitivity and reduce body fat that subsequently preventing metabolic syndrome, diabetes, and obesity [46].

The strength of our work is that this systematic review and meta-analysis is the first study that comprehensively pooled the results of available literature regarding the role of probiotics and selenium co-supplementation on lipid profile and glycemia indices. Our findings have research and clinical implications and can be used by clinicians and health practitioners. However, some limitations should be considered while interpreting the results. The small number of the included studies (*n* = 5) may diminish the precision of pooled effect estimates. Substantial heterogeneity should also be considered that can reduce the generalizability of our findings. Participants’ characteristics (i.e., sex, age, ethnicity, genetic profile, and health status), sample size, study duration, dose, and strain of probiotics are among the possible sources of heterogeneity. However, due to the small number of the included studies, we were unable to run a sub-group analysis to find the sources of heterogeneity.

## Conclusion

The findings of the current systematic review and meta-analysis suggested that co-supplementation with probiotics and selenium may benefit adults in terms of FPG, insulin, HOMA-IR, QUICKI, TC, LDL-C, and TG. However, due to the small number of included studies, further trials are needed to further investigate this issue. Moreover, further studies are needed with better methodology to compare the synergistic effects of selenium and probiotic co-supplementation to supplementation with probiotic or selenium alone.

## Supplementary Information

Below is the link to the electronic supplementary material.

Supplementary file1 (DOCX 31 KB)

## Data Availability

The datasets generated during and/or analysed during the current study are available from the corresponding author on reasonable request.
